# 33. Evaluating the Safety and Effectiveness of a Non-Severe Community-Acquired Pneumonia Pharmacist Pathway

**DOI:** 10.1093/ofid/ofab466.235

**Published:** 2021-12-04

**Authors:** Kelly Sylvain, Jennifer Polenska, James Levin

**Affiliations:** SSM Health St. Mary’s Hospital - Madison, Madison, Wisconsin

## Abstract

**Background:**

One of the main roles of the SSM Health WI Regional Antimicrobial Stewardship Program is to create infection treatment pathways based on the Infectious Diseases Society of America (IDSA) practice guidelines. Treatment pathways are used to guide provider prescribing of antimicrobials for disease states such as community-acquired pneumonia (CAP). The objective of this study was to evaluate the safety and effectiveness of a non-severe CAP pharmacist pathway based on the updated IDSA and American Thoracic Society 2019 CAP practice guideline.

**Methods:**

A retrospective chart review was performed on all patients placed on the non-severe CAP pharmacist pathway at SSM Health St. Mary’s Hospital in Madison, WI from September 2020 through April 2021. Patients who initially started on the pathway were removed if they met prespecified criteria (Table 1). The primary outcome in this study was 30-day respiratory-related readmission rate. Secondary outcomes included average total length of antibiotic therapy, pharmacist interventions [intravenous (IV) to oral (PO) conversion, antibiotic de-escalation (including discontinuation of azithromycin with negative legionella urinary antigen), duration of therapy], and 30-day all-cause readmission rate.

Table 1. Criteria for Removal from the Pathway

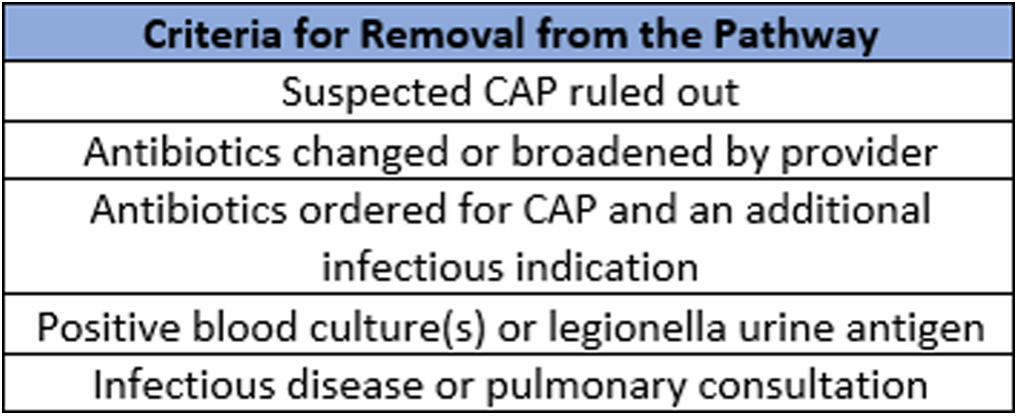

Figure 1. Pharmacist Interventions

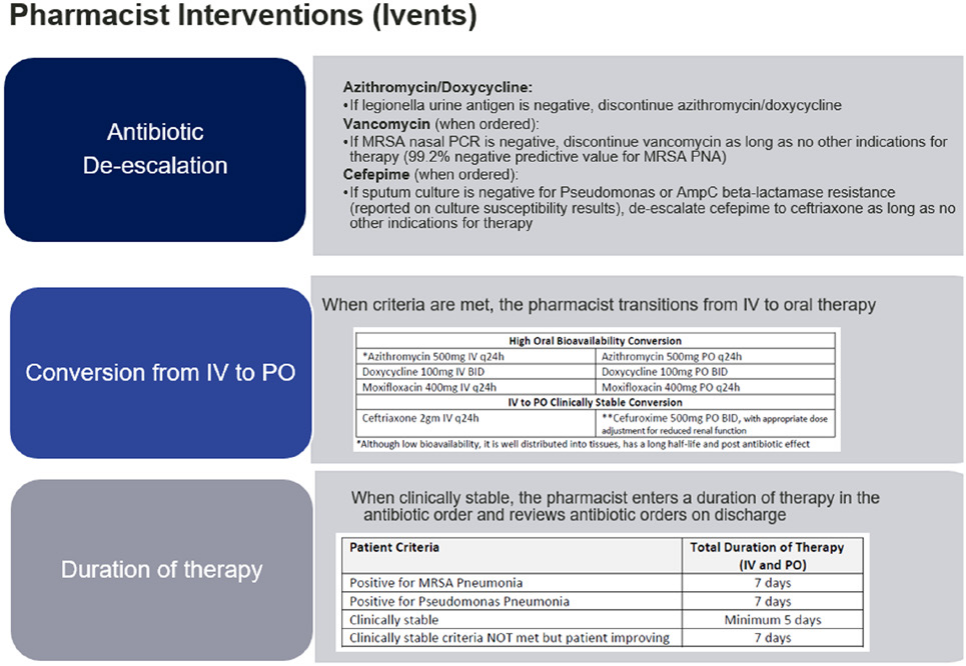

**Results:**

A total of 119 patients were initiated on the non-severe CAP pharmacist pathway, of which 47 patients (40%) completed the pathway and 72 patients (60%) were removed from the pathway. Of the 47 patients who completed the pathway, there were no respiratory-related readmissions with a 30-day all-cause readmission rate of 6.4% (N=3/47). The average total duration of beta-lactam therapy was 6.8 days and the average total duration of macrolide therapy was 1 day due to de-escalation with a negative legionella urinary antigen result. A total of 61 pharmacist-driven interventions were completed [IV to PO conversion (N=15), de-escalation (N=27), and duration of therapy (N=19)].

Table 2. Summary of Results

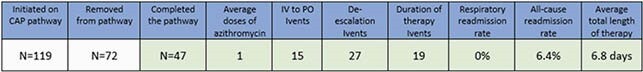

**Conclusion:**

The findings of this study suggest that implementation of a non-severe CAP pharmacist pathway is safe and effective. No readmissions were related to non-severe CAP management and pharmacists completed guideline-driven interventions related to antimicrobial de-escalation, IV to PO conversion, and duration of therapy.

**Disclosures:**

**All Authors**: No reported disclosures

